# Metabolomics and Molecular Approaches Reveal Drought Stress Tolerance in Plants

**DOI:** 10.3390/ijms22179108

**Published:** 2021-08-24

**Authors:** Manoj Kumar, Manish Kumar Patel, Navin Kumar, Atal Bihari Bajpai, Kadambot H. M. Siddique

**Affiliations:** 1 Institute of Plant Sciences, Agricultural Research Organization, Volcani Center, Rishon LeZion 7505101, Israel; 2 Department of Postharvest Science of Fresh Produce, Agricultural Research Organization, Volcani Center, Rishon LeZion 7505101, Israel; patelm1402@gmail.com; 3 Department of Life Sciences, Ben-Gurion University, Be’er Sheva 84105, Israel; navinmsbc@gmail.com; 4 Department of Botany, D.B.S. (PG) College, Dehradun 248001, Uttarakhand, India; dratalbajpai@gmail.com; 5 The UWA Institute of Agriculture, and UWA School of Agriculture and Environment, The University of Western Australia, Perth, WA 6001, Australia

**Keywords:** crop improvement, drought stress, drought tolerance, genetic engineering, metabolomics, primary metabolites, secondary metabolites

## Abstract

Metabolic regulation is the key mechanism implicated in plants maintaining cell osmotic potential under drought stress. Understanding drought stress tolerance in plants will have a significant impact on food security in the face of increasingly harsh climatic conditions. Plant primary and secondary metabolites and metabolic genes are key factors in drought tolerance through their involvement in diverse metabolic pathways. Physio-biochemical and molecular strategies involved in plant tolerance mechanisms could be exploited to increase plant survival under drought stress. This review summarizes the most updated findings on primary and secondary metabolites involved in drought stress. We also examine the application of useful metabolic genes and their molecular responses to drought tolerance in plants and discuss possible strategies to help plants to counteract unfavorable drought periods.

## 1. Introduction

Drought stress (DS) negatively affects plant morphological, physiological, and biochemical processes, which decrease photosynthesis [1], impair cell elongation and division [2], and reduce cell turgor pressure [3]. Drought stress also inhibits nutrient uptake and affects gene expression, yield, and quality of crop plants [4,5]. Metabolites play an essential role in plant growth and development. Under stress conditions, metabolites are involved in cell signaling, energy storage, membrane formation and scaffolding, and whole-plant resource allocation [6]. Various abiotic stresses, including drought, disturb plant metabolism through metabolic enzyme inhibition, substrate shortage, excess demand for specific compounds, or a combination of these and many other factors. Thus, the metabolic network must be reconfigured to maintain essential metabolism, and acclimate by adopting a new steady-state in light of the prevailing stress conditions [7]. The induction of primary or secondary metabolites under drought stress can regulate the turgidity and stiffness of cells and tissues, redox homoeostasis, ion transport, and enzyme activity [8,9]. These metabolites play an important role in connecting plant genotypes and phenotypes [10,11].

Metabolomics is an effective tool for garnering comprehensive information on metabolite profiling and metabolic network analysis. It also imparts knowledge about identified and unidentified metabolites. Several reports have contributed to the recent understanding of metabolite regulation in many plant species in response to different environmental stresses, including drought, salt, heat, cold, and light stress [7,12,13]. Metabolite profiling approaches have been widely used to characterize the molecular responses to DS in plants and evaluate metabolite levels in a particular metabolite class or pathway [14,15]. It includes various analytical approaches for identifying different classes of metabolites through gas chromatography-mass spectrometry (GC-MS), liquid chromatography-mass spectrometry (LC-MS), nuclear magnetic resonance (NMR), high-performance liquid chromatography (HPLC), and capillary electrophoresis-mass spectrometry (CE-MS) in various plant species under DS [16,17,18] (Table 1).

The detection of molecular traits that differ in response to stress events is challenging. Metabolic profiling could be used to characterize molecular traits implicated in the stress response, providing valuable information for breeding programs [19]. Certainly, metabolomics is a powerful technique for identifying biological or physiological responses to environmental changes, particularly when combined with other omics approaches, such as transcriptomics and proteomics [20,21,22]. Metabolomics is used to identifying and/or quantifying primary and secondary stress-responsive metabolites in plants under abiotic stress, including DS [23]. This review summarizes the most updated findings on metabolomics and molecular approaches to reveal drought stress tolerance in plants. 

## 2. Metabolomics and Its Application in Drought Tolerance of Plants

Environmental stresses, such as drought, salinity, and high temperatures, can trigger hyper-accumulation of a vast array of metabolites in plants [24,25]. Plant secondary metabolites (SMs) are derivatives of primary metabolites (PMs) produced by plants to fight a variety of unfavorable physiological changes induced due to stressors [26,27]. Drought is one of the most significant environmental stresses on agricultural production worldwide [28]. In plants, DS adaptation is a complicated biological process that involves dynamic trends in metabolite composition and gene expression [29]. Plant tolerance to DS is typically determined by their ability to maintain an appropriate level of primary and secondary metabolic processes and defense responses [25]. Metabolomic analysis can investigate and recognize key differences between DS-tolerant and DS-sensitive plant species/genotypes and connect links between genotypic and phenotypic changes in plants during DS [30]. Two main methods (non-targeted and targeted) are used to understand metabolic reprogramming in plants under abiotic stress [31,32,33]. Non-targeted metabolomics provides an overview of the most abundant metabolites in plants under various environmental stresses. Targeted metabolomics detects, estimates, and analyzes known metabolites in plants under various environmental stresses [34,35]. Therefore, metabolomics studies can reveal the important role for metabolic reprogramming, including regulation and accumulation of PM and SM levels in plants under DS and biotechnological applications for DS management of agricultural crop plants [36,37].

### 2.1. Drought-Induced Adjustment of Primary Metabolites

**Figure 1:**
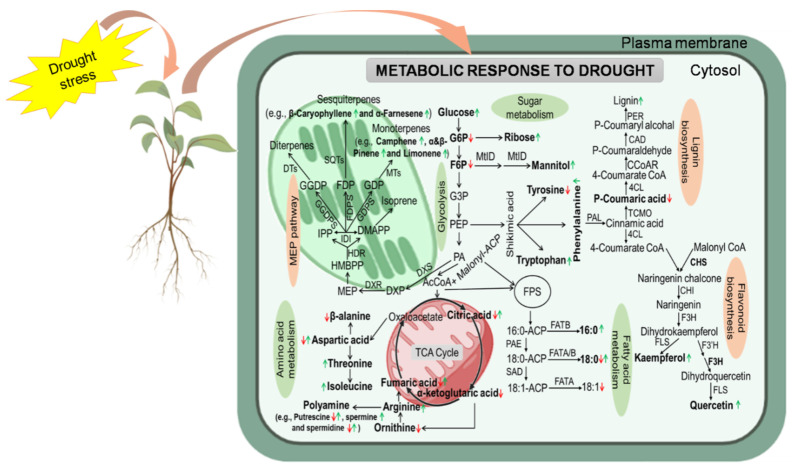
A schematic representation of metabolic response to drought stress. Primary metabolites (PMs) and secondary metabolites (SMs) are reprogrammed in plant cells to maintain osmotic balance and activate various primary and secondary metabolic pathways (green and orange circles, respectively) to survive under DS. Metabolites with an important role in DS are highlighted in bold, and their responses are depicted with green arrow (increased level), red arrow (decreased level), and green and red arrows (increased/decreased levels). ACP, acyl carrier protein; 4 CL, 4-coumarate-CoA ligase; AcCoA, acetyl-CoA; CAD, cinnamyl alcohol dehydrogenase; CCoAR, cinnamoyl-CoA reductase; CHI, chalcone isomerase; CHS, chalcone synthase; DMAPP, dimethylallyl diphosphate; DTs, diterpene synthase; DXP, 1-deoxy-D-xylulose-5-phosphate; DXS, 1-deoxy-D-xylulose 5-phosphate synthase; FAT A/B, fatty acyl-ACP thioesterase A/B; F3H, flavanone 3-hydroxylase, F3’H, flavonoid 3‘-hydroxylase; F6P, fructose 6-phosphate; FDP, farnesyl diphosphate; FDPS, farnesyl diphosphate synthase; FLS, flavonol synthase; G3P, glyceraldehyde 3-phosphate; G6P, glucose 6-phosphate; GDP, geranyl diphosphate; GDPS, geranyl diphosphate synthase; GGDP, geranyl geranyl diphosphate; GGDPS, geranyl geranyl diphosphate synthase; HDR, 1-hydroxy-2-methyl-2-(E)-butenyl 4-diphosphate reductase; HMBPP, 1-hydroxy-2-methyl-2-(E)-butenyl 4-diphosphate; IDI, isopentenyl diphosphate isomerase; IPP, isopentenyl diphosphate; MtID, mannitol-1-phosphate dehydrogenase; MEP, 2-C-methyl-D-erythritol-4-phosphate; MTs, monoterpene synthase; PAE, palmitoyl-ACP elongase; PA, pyruvic acid; PAL, phenylalanine ammonia-lyase; PEP, phosphoenolpyruvate; PER, peroxidase; SAD, stearoyl-ACP desaturase; SQTs, sesquiterpene synthase; TCA, tricarboxylic acid; TCMO, trans-cinnamate 4-monooxygenase. Figure adapted from images created with BioRender.comto draw the proposed model (https://app.biorender.com/biorender-templates (accessed on 18 June 2021)).

Drought stress directly affects plant metabolism, resulting in profound changes in biosynthesis and transport of PMs and SMs [38,39]. Primary metabolites are important for the proper development of plant cells and directly implicated in plant growth processes, photosynthesis, and respiration [25,40]. They include sugars, polyols, amino acids (AAs), and lipids that allow plants to acclimatize and recover from DS [41] (Figure 1; Table 1). 

#### 2.1.1. Carbohydrates

Sugars play an important role in carbon resource allocation and plant growth [42]. Sugars (glucose, sucrose, fructose, ribose, raffinose, erythronate, and xylonate) and pyruvate, a glycolysis intermediate, increased in roots under DS [43]. Myoinositol content decreased under dehydration stress in *Arabidopsis* [29]. Similarly, trehalose levels declined in *Zea mays* [44] and *Oryza sativa* [45]. Sugars (glucose, galactose, fructose, and maltose) and sugar alcohols (arabitol and galactitol) accumulated in *Lotus japonicus* during DS [46]. Ogbaga et al. [47] studied two drought-contrasting sorghum (*Sorghumbicolor*) cultivars (Samsorg 17 and 40) under DS and reported significant upregulation of sugars (fructose, cellobiose, galactose, lactose, and sedoheptulose) and sugar alcohols (myoinositol, ribitol, and xylitol) in Samsorg 17, relative to Samsorg 40, in response to DS. *Nicotiana tabacum* leaves and roots showed different metabolic responses under DS. In leaves, glucose-6-phosphate and fructose-6-phosphate decreased and mannitol increased [48]. In roots, galactinol and myoinositol increased initially but decreased later, and mannitol and trehalose increased [48]. In *N*. *tabacum*, 4-hydroxy-2-oxoglutaric acid was undetectable in leaves but increased 20-fold in roots during the initial hours (between 1 and 2 h) of drought stress and 70-fold after four hours. This indicates that *N*. *tabacum* under DS accumulates 4-hydroxy-2-oxoglutaric acid, which is subsequently broken down into pyruvate and glyoxylate when water becomes available [48]. Yang et al. [49] analyzed drought-tolerant (Lo964) and drought-sensitive (B73) inbred lines of *Z. mays* at 7 and 14 days after drought imposition (DAI). Under DS, carbohydrates such as sucrose, fructose, galactinol, raffinose, and ketose increased in B73 and decreased in Lo964. Components of the TCA cycle, such as citrate, succinate, α-ketoglutarate, and fumarate, decreased in Lo964 at 7 or 14 DAI, while isocitrate and citrate increased in B73 at 7 DAI [49]. In *Z*. *mays*, glutathione and urea cycles as well as carbohydrate and lipid metabolism play a key role in osmoprotection, membrane maintenance, and antioxidant protection during DS [49]. Four metabolites (ornithine, arginosuccinate, arginine, and citrulline) were downregulated in B73 under DS at 7 DAI, but exhibited different patterns in Lo964 [49]. Obata et al. [44] analyzed myoinositol and glycine using GC-MS in leaf blade tissue of *Z. mays* under DS and found significant correlations between myoinositol and glycine levels and grain yield.

#### 2.1.2. Amino Acids

Amino acids are essential metabolites in plants for protein synthesis and cellular function [50]; they also function as osmolytes to balance cellular osmotic potential and as scavengers of reactive oxygen species (ROS) generated in plants under DS. The aromatic AAs (phenylalanine, tryptophan and tyrosine) are important components of protein synthesis in plants and serve as precursors for several secondary metabolites that are essential for plant growth [51]. Differential accumulation of metabolites occurred in two drought-contrasting chickpea (*Cicer arietinum* L.) genotypes using the UPLC-HRMS-based untargeted metabolic profiling approach [52]. Under DS, other PMs (e.g., proline, arginine, histidine, isoleucine, and tryptophan) accumulated in the leaves of the tolerant chickpea variety, while alanine, α-ketoglutaric acid, GABA, choline, tyrosine, glucosamine, adenosine, guanine, and aspartic acid decreased in both genotypes [52]. Active aromatic AAs may function as a secondary source of energy and have been implicated in stress tolerance in chickpea. Aromatic AAs (phenaylalanine and tyrosine) are precursors of several SMs, including indole acetate, and lignin in the shikimate pathway, which play an important role in stress tolerance [53,54]. Many essential metabolites, such as sugars, AAs, and GABA, increased in wheat (*Triticum aestivum*) exposed to DS compared to control plants [55]. Similarly, increased levels of proline, methionine, lysine, and arginine contents occurred in drought-tolerant and drought-sensitive wheat genotypes in response to DS [56] (Table 1). Sanchez et al. [46] reported that aspartic acid, glutamic acid, and phosphoric acid decreased during DS in *L*. *japonicus*. Alanine and glutamine contents decreased in drought-tolerant and drought-sensitive soybean *(Glycine max* L.) genotypes under DS, whereas GABA decreased in the tolerant genotype and aspartic acid content increased in the sensitive genotype [57].

Significant metabolite accumulation was detected in one or more organs of *Hordeum vulgare* under DS [58]. Valine was significantly upregulated in the fifth leaf, awn, and lemma, and proline accumulated in all organs (fifth leaf, awn, lemma, and palea) during DS [58]. Phenylalanine was significantly upregulated in the lemma and fifth leaf, and glycine, isoleucine, and threonine accumulated in the fifth leaf and awn during DS. Moreover, sugars accumulated in the spike organs (awn, lemma, and palea) during DS [58]. Amino acids and osmolytes maintain turgor pressure and protect cellular processes through ROS scavenging [58]. De Miguel et al. [43] reported that the concentrations of sugars and AAs increased significantly in aerial organs and roots of *Pinus pinaster* under DS. The relative increase in AAs is related to a process of protein degradation under stress [59], as measured by the increase in asparagine levels in roots and stems of *P*. *pinaster* but not needles under DS [43]. Amino acids from the aspartate and glutamate families increased in aerial organs and roots, whereas aromatic AAs (phenylalanine) were increased in roots of *P. pinaster* during DS [43]. Plants under DS accumulate various osmolytes, such as carbohydrates, AAs, and glycine betaine, which play a key role in regulating osmotic potential, controlling ion transport and cell turgor pressure, and stabilizing cell membranes [60,61]. Thus, changes in metabolite contents under DS indicate that regulation of primary metabolism is crucial for DS tolerance in crop plants (Figure 1).

#### 2.1.3. Lipids and Fatty Acid

Lipids are cellular macromolecules with structural, energy storage, and signalling roles in plant biological systems [62]. Lipids act as signaling mediators [63,64] to mitigate the negative impacts of environmental stressors [65,66]. Plant lipids principally include glycerolipids (e.g., phospholipids, galactolipids, sphingolipids, triacylglycerols) and extracellular lipids (e.g., suberin, cutin, and waxes). Sanchez-Martin et al. [67] profiled different classes of lipids, including polar lipids (PLs), monoacylglycerols (MAGs), diacylglycerols (DAGs), and triacylglycerols (TAGs) and free fatty acids (FFAs) in drought-tolerant (cv. Patones) and drought-sensitive (cv. Flega) oat cultivars differing in their response to drought stress. Saturated FAs, particularly palmitic acid in the DAG and TAG fractions, increased in drought-sensitive cv. Flega. In contrast, drought-tolerant cv. Patones was characterized by the early induction of signaling-related fatty acids and lipids, such as linolenic acid and DAGs [67]. Moradi et al. [68] examined lipid profiling in drought-tolerant and drought-sensitive thyme plants under prolonged drought stress and found that lipid components decreased in sensitive plants but increased in tolerant plants. They proposed that combining lipid profiling with physiological parameters represented a promising tool for investigating the mechanisms of plant response to DS at the non-polar metabolome level [68]. The composition of lipid components changes under DS. The lipid contents in *A*. *thaliana* leaves decreased progressively in response to DS. However, the lipid content of highly dehydrated leaves quickly increased after rehydration [69]. 

Drought elevated the levels of major lipid components, indicating enhanced lipid biosynthesis and/or reduced lipid degradation [68]. Stress-induced changes in the lipid profile cause membrane lipid remodeling and activation of plant defense mechanisms against biotic and abiotic stresses, including drought [70,71]. Zhang et al. [72] used lipidomic analyses to investigate the responses of cutin monomers and cuticular waxes to drought stress in drought-tolerant (cv. Kangsi) and drought-sensitive (cv. Hongyingzi) cultivars of *Sorghum bicolor*. In drought-tolerant cv. Kangsi, drought increased cutin content by 41.3%, alkanoic acid level by 72.6%, and 2-hydroxyacid content by 117.8%, but had no effect on drought-sensitive cv. Hongyingzi. Drought increased total wax coverage in cv. Hongyingzi but decreased it in cv. Kangsi [72]. Gundaraniya et al. [73] reported that drought stress increased the accumulation of saturated FAs (stearic acid) in leaves of a drought-tolerant peanut genotype, whereas 8, 11-octadecadienoic acid accumulated in roots of a drought-sensitive peanut genotype. Several FAs were detected in purslane leaves under DS, including palmitic acid, linolenic acid, linoleic acid, oleic acid, stearic acid, arachidic acid and behenic acid [74]. Drought treatments significantly increased palmitic acid content, but decreased stearic acid and oleic acid contents compared to the control [74]. FAs and lipids are involved in growth, development and responses to biotic and abiotic stresses for acclimation [75].

### 2.2. Drought-Induced Adjustment of Secondary Metabolites

In response to various environmental stresses, including drought, plants produce SMs [60,76]. The biosynthesis of SMs is regulated by environmental factors and has a chief role in protecting plants against environmental stresses [25,77]. Secondary metabolites are categorized into three main groups, including nitrogen-containing metabolites such as alkaloids (e.g., cyanogenic glycosides, glucosinolates, etc), phenolic compounds (e.g., tannins, flavonoids, lignins), and terpenes (e.g., terpenoids or isoprenoids) [25,78] (Figure 1; Table 1). Secondary metabolite pathways include mevalonic acid, shikimic acid, phenylpropanoic acid, and methylerythritol phosphate [79,80]. Drought induces oxidative stress in plants, which produces reactive oxygen species (ROS). Secondary metabolites mainly scavenge ROS to protect plant cells from lipid peroxidation and play an important role in other defense-related activities [81]. Key enzymes of the phenylpropanoid pathway, including phenylalanine ammonia-lyase (PAL), 4-coumaroyl CoA ligase (4CL), and coumarate-4-hydroxylase (C4H), were implicated under stress conditions [82] (Figure 1). Additionally, drought-induced volatile compounds can warn tissues to activate DS-mitigating functions in plants [83] (Figure 1). The production of SMs may alter in response to DS in plants. Secondary metabolite accumulation enhances stress tolerance by modulating physiological and biochemical parameters in plants [84].

#### 2.2.1. Phenolics

Plant phenolics, such as flavonoids, tannins, coumarins, and lignins, are a significant family of SMs [85]. Flavonoids are a class of naturally occurring phenolic compounds that include flavones, isoflavones, flavanones, flavonols, chalcones, proanthocyanidins, and anthocyanidins [86]. The phenolics contents in *Hypericum brasiliense* increased under water-deficit conditions [87]. Quercetin was upregulated under water-deficit conditions, while rutin was upregulated in response to hypoxia and DS [87]. Increased flavonol content in plants has a role in the protection against ROS [88]. Under DS, flavonoids and phenolics contents increased in *Achillea* species [89]. In *Achillea pachycephala*, DS increased luteolin-7-O-glucoside and decreased apigenin-7-O-glucoside content [90]. Similarly, DS significantly elevated phenolic compounds such as salicylic acid, ferulic acid, and 4-hydroxycinnamic acid drought-tolerant *G. max* compared to the common wild-type [91]. Salicylic acid can stimulate the plant antioxidant system, improving cell metabolic activity [58]. Ferulic acid is a strong antioxidant that can improve cell membrane integrity under various abiotic stresses [92,93]. During drought, luteolin levels increased in leaves of chrysanthemum cultivars, while apigenin levels decreased or remained unchanged [94]. Drought stress decreased flavonoid and phenolic compound contents, specifically p-coumaric acid, chlorogenic acid, and rutin, in the flower extract of safflower (*Carthamus tinctorius* L.) but increased caffeic acid, ellagic acid, vanillic acid, and quercetin contents [95]. In the same study, DS decreased ferulic acid, vanillic acid, p-coumaric acid, apigenin, quercetin, rutin, and luteolin contents in the seed extracts of *C. tinctorius* but increased caffeic acid, ellagic acid, chlorogenic acid, and gallic acid contents [95]. Changes in the amount of flavonoid compounds during abiotic stress could be linked to ROS generation due to the role of flavonols in plant defense [88].

Long-term progressive DS significantly reduced total isoflavone content in *G. max* seeds [96]. Drought stress increased endogenous SM contents in various medicinal plants. For example, quercetin, rutin, and betulinic acid increased in *Hypericum brasiliense* and *Artemisinin artemisia* under DS [97]. In another study, the level of phenolic compounds increased in *Trachyspermum ammi* under DS [98]. Flavonoids increased in *Glechoma longituba* under DS [99]. In *Sesamum indicum* L, DS increased flavonoid, phenolic, and polyphenolic contents, while sesamin, oil, and quercetin contents decreased [100]. Plant polyphenols, such as phenolics and flavonoids, are biosynthesized in plants via different pathways [89]. Phenolic acids increase under DS due to the lignification of cell walls and production of AAs, mainly phenylalanine and tyrosine, to maintain cell osmotic equilibrium [101]. Under DS, factor OsC1-MYB upregulated transcripts of flavonoid biosynthesis-related genes *OsDFR* and *OsANS* in *O. sativa* [102]. Drought stress enhanced the levels of total phenolics, flavonoids, and anthocyanins in *T*. *aestivum* [103]. Quantitative real-time PCR analysis revealed that *TaCHS*, *TaCHI*, *TaFLS*, *TaF3H*, *TaFNS*, *TaANS,* and *TaDFR* expression levels increased under DS in two *T*. *aestivum* cultivars, Aikang 58 (AK) and Chinese Spring (CS) [103].

Lignins are a high-molecular-weight natural phenolic polymer that play an important role in cell wall formation of plants [104]. Lignin accumulation plays a key role in plant biotic and abiotic stress tolerance [105,106]. Drought stress significantly enhanced the expression of lignin biosynthetic gene *cinnamoyl CoA reductase (CCR)* in *Leucaenal eucocephala* seedlings. The accumulation of CCR protein is associated with drought tolerance [107]. Overexpression of the *SiMYB56* gene from *Setaria italica* enhanced drought tolerance in transgenic rice plants by lowering MDA content and increasing lignin content under drought [108]. Similarly, overexpression of the *VlbZIP30* gene from *Vitis labrusca* enhanced DS tolerance in transgenic grapevine plants by maintaining photosynthesis rate and increasing leaf lignin content under drought conditions [109]. 

#### 2.2.2. Trepenes and Polyamines

Terpene synthases are the main gatekeepers engaged in terpene biosynthesis. Drought stress upregulates terpenes (e.g., monoterpene, diterpenes, and sesquiterpene) and their biosynthesis [110,111]. Diterpenes provided drought tolerance in *Salvia officinalis* plants by inducing the ROS system [112]. Vallat et al. [113] studied the effects of relative humidity on apple (*Malus domestica*), revealing that low humidity stimulates the production of terpenes such as camphene, α-pinene, β-pinene, β-caryophyllene, limonene, and α-farnesene. Exogenous application of salicylic acid increased the level of terpenes in lemongrass (*Cymbopogon flexuosus* Steud. Wats.) under DS [114]. Naturally occurring SMs (triterpenoid, oleanolic acid, and betulin) increased in *Betula platyphylla* under mild and mild/severe DS [115]. *Craterostigma plantagineum* plants exposed to DS for 4 days gradually increased the level of polyamines spermine (Spm) and spermidine (Spd) up to 8-fold and 3-fold, respectively, relative to control plants, and the amount of putrescine (Put) decreased [116]. Put and Spd levels were significantly reduced under drought stress, whereas Spm levels were either maintained or slightly increased in *O. sativa* cultivars [117]. While Put was the main polyamine under control conditions, Spm became the dominating polyamine during drought stress, however there were no significant associations between polyamine concentration and drought tolerance cultivar [117]. The findings discussed above and additional studies in Table 1 show that reprogramming PMs and SMs in plants is an adaptive response to DS tolerance (Figure 1).

**Table 1:** Key metabolites involved in various plant species under drought stress.

Plant Species	Methods of Analysis	Tissue	Key Metabolites Involved in Drought Tolerance	References
Monocots				
*Avena sativa*	GC	Leaves	Lipids: Monoacylglycerols (MAGs), diacylglycerols (DAGs), and triacylglycerols (TAGs) and free fatty acids (FFAs)	[67]
			FA: Palmitic acid, linolenic acid	
*Brachypodium distachyon*	GC/MS	Leaves	CH: Glucose, glycerol, mannobiose, maltose, sucrose, galactose	[118]
			AA: Norvaline	
*Hordeum vulgare*	HPLC-DAD-MSn	Leaves	SM: Flavone glycosides, chlorogenic acids, caffeoyl-hexose, sinapoyl-hexoses, feruloyl-hexose, hydroxycinnamic acids	[93]
*H. vulgare*	GC-MS	Awns, kernels	CH: Galactinol, mannitol	[119]
			OM: Isocitric acid, α-ketoglutaric acid	
*H. vulgare*	GC-MS	Grain	CH: Raffinose, mannitol, myoinositol, putrescine,	[120]
			AA: Pyroglutamic acid	
*H. vulgare*	GC-MS-EI	Fifth leaf, palea	AA: Proline, glutamine, threonine, glycine, aspartate, serine, aromatic amino acids	[58]
*Oryza sativa*	GC/EI-TOF-MS	Leaves	AA: Glutamate, arginine, proline	[117]
			PA: Spermidine, putrescine, spermine	
			OM: GABA	
*O. sativa*	GC/MS	Leaf blades	AA: Serine, asparagine, threonine	[121]
*Triticum aestivum*	GC-TOF-MS	Shoots	CH: Sucrose, mannose, fructose	[13]
			AA: Proline	
			OM: Malic acid	
*T. aestivum*	GC/MS	Flag leaves	AA: Glutamine, methionine, lysine, asparagines, serine	[122]
*T. aestivum*	GC-MS	Roots, leaves	AA: Valine, tryptophan	[123]
			OM: Malic acid, fumaric acid, citric acid,	
Seven Triticeae species	GC-MS	Roots, leaves	CH: Sucrose, trehalose, mannitol, maltose	[124]
			AA: Proline, glutamate, alanine, glycine, asparagines, methionine, threonine, phenylalanine, homocysteine, serine, valine, tyrosine	
			OM: Succinate, citrate, aspartate, gluconate, glutathione	
*Zea mays*	GC/MS	Leaf blades	AA: Glycine, myoinositol	[44]
*Z. mays*	^1^H-NMR	Leaves	AA: Alanine	[125]
			Lipids: Triacylglyceride	
			OM: Malate, glutamate, formate	
Dicots				
African eggplant	GC-MS	Leaves	CH: Fructose, sucrose	[126]
			AA: Proline, glutamate	
			OM: Tricarboxylic cycle metabolite	
*Arachis hypogaea*	GC-MS	Nodules	CH: Trehalose	[127]
			AA: Proline	
			OM: GABA	
*A*. *hypogaea*	GC-MS	Leaves, roots	CH: Glucose D-ribose, D-mannitol, D-xylopyranose, xylonic acid, α-D-glucopyranose, 2-deoxyribose, L-manopyranose, myo-inositol, galactosoxime, D-fructose, D-turanose, malic acid, succinic acid, 2 butenedoic acids, 2-deoxyribose, myo-inositol	[73]
			FA: Stearic acid, pentadecanoic acid, 8,11-octadecadienoic acid, palmitic acid, pentadecanoic acid	
*Craterostigma* *plantagineum*	HPLC	Leaves	PAs: Putrescine, spermine, spermidine	[116]
*Cicer arietinum*	UPLC-HRMS	Leaves	AA: l-proline, l-arginine, l-histidine, l-isoleucine, tryptophan	[52]
			OM: Allantoin	
*Glycine max*	^1^H-NMR, ^1^H-^1^H TOCSY	Leaves, nodules	CH: Myoinositol, pinitol	[57]
			AA: Glutamine	
			OM: GABA, allantoin	
*G. max*	NMR	Leaves, roots	CH: Sucrose	[128]
			AA: Alanine	
			OM: Succinate, citrate, acetate	
*G. max*	GC-MS	Leaves	SM: 5-methoxytryptamine, 4-hydroxycinnamic acid, ferulic acid, salicylic acid	[91]
			OM: Fluorine	
Lentils	GC/EI-TOF-MS	Cotyledons, radicles, shoots	PAs: Putrescine, cadaverine	[129]
			CH: Erythronic acid	
			OM: Isocitric acid, nicotinic acid	
*Nicotiana tabacum*	GC/MS, LC/MS	Leaves, roots	CH: Mannitol, trehalose, myoinositol, galactinol	[129]
			OM: GABA	
*Nigella sativa*	GC	Seeds(10 black cumin genotypes)	FA: Stearic acid, palmitic acid, oleic acid, linoleic acid, linolenic acid, myristic acid, arachidic acid	[130]
*Portulaca oleracea*	GC	Leaves	FA: Palmitic acid, linolenic acid, linoleic acid, oleic acid, stearic acid, arachidic acid, behenic acid	[74]
*Vigna unguiculata*	GC-TOF	Seeds	CH: Galactinol	[131]
			AA: Proline	
			SM: Quercetin	
*V. unguiculata*	GC-TOF	Leaves	CH: Rhamnose, raffinose	[132]
*Vitis vinifera*	SPME-GC-MS	Leaves	SM: Quercetin-3-O-glucoside, kaempferol-3-O-glucoside	[133]
			OM: Citric acid, 2-methyl-butanal phenylacetaldehyde	

AA, amino acid; CH: carbohydrate; EI, electrospray ionization; FA, fatty acid; GABA, γ-aminobutyric acid; GC-MS, gas chromatography-mass spectrometry; HPLC-DAD-MS, high-performance liquid chromatography coupled with diode-array detection and multiple-stage mass spectrometry; LC-MS, liquid chromatography-mass spectrometry; ^1^H-NMR, nuclear magnetic resonance; OM, other metabolites; PAs, Polyamines; SM, secondary metabolites; SPME-GC-MS, solid phase micro extraction-gas chromatography mass spectrometry; TOCSY, total correlation spectroscopy; TOF, time-of-flight; UPLC-HRMS, ultra-performance liquid chromatography-high-resolution mass spectrometry.

## 3. Metabolomic and Molecular Responses to Drought

Metabolic regulation is the key mechanism implicated in the safeguarding of cell osmotic potential during abiotic stress. The metabolite profiling approach has been widely used to characterize molecular responses of plants under abiotic stress [134]. Apart from its importance for cell function, water is an important component of plants due to its undeviating involvement in metabolite transportation and essential nutrients to various plant parts. Inaccessibility of sufficient water or higher transpiration rates enhances DS and changes metabolite production [83]. Drought tolerance strategies of plants comprise numerous biological mechanisms at the cell, organ, and whole-plant levels when stimulated at different phases of plant growth. Drought stress affects plants at several levels, including the molecular level [135], increasing the accumulation of drought-related proteins and metabolites [136]. Several molecular pathway cascades, including perception of water deficiency, activation of signaling network, and transcriptional, metabolic, and regulatory element responses improve plant resistance to DS [137]. Molecular mechanisms of the drought response are strongly governed by regulatory elements, such as transcription factors (TFs) and protein kinases. Transcription factor families, such as MYB, NAC, bZIP, AP2/ERF, and AREB/ABF, regulate stomatal movement and the expression of drought-responsive genes upstream or downstream of a metabolic pathway [138,139]. 

The molecular response to drought stress is a multi-genic trait controlled by many genes. Several genes related to DS at the transcriptional level have been investigated in microarray and real-time polymerase chain reaction (RT-PCR) studies [134,135,136,137,138,139,140,141,142]. Functional validation revealed that these genes protect against dehydration stress through stress perception, signal transduction, and transcriptional regulatory networks responses to drought tolerance [143,144]. Therefore, understanding molecular responses to drought tolerance can provide insights for enhancing drought tolerance in sensitive plant species. Significant efforts have been made to explore the molecular mechanisms used by plants to cope with DS. Plants respond to DS by reprogramming their transcriptional, proteomic, and metabolic pathways to protect cells from stress-mediated damage [144,145,146]. Primary metabolites, such as glucose, sucrose, and trehalose, function as signal molecules to regulate gene expression involved in plant growth and the stress response [147]. Drought stress elevates ROS production in diverse cellular compartments, particularly chloroplasts and mitochondria, which is controlled by an adaptable and cooperative antioxidant system that balances intracellular ROS levels and sets the redox status of plant cells [148]. 

## 4. Genetic Engineering of Metabolic Genes to Improve Drought Tolerance in Plants

The genetic engineering approach is widely used to enhance plant tolerance to various environmental stresses, including drought, by engineering candidate genes for crop improvement [39,149,150,151]. Drought stress can seriously impact plant growth, photosynthesis, water relations, yield, pigment content, and membrane integrity [152]. Plants have evolved various interconnected signaling networks to regulate drought-responsive genes to produce various classes of proteins, including transcription factors, enzymes, molecular chaperones, and other functional proteins, for drought tolerance [153]. Developing drought-tolerant plants using the genetic engineering approach requires identifying key genetic determinants underlying DS tolerance in plants and introducing metabolic genes into crops for expression. Drought-responsive genes are involved in signaling cascades, transcriptional regulation (e.g., transcription factors and protein kinase/phosphatase), and functional proteins that protect cell membranes [154]. Other proteins, such as antioxidants, osmotin, late embryogenesis abundant proteins, and proteins associated with the uptake and transport of water and ions, such as aquaporins and sugar transporters, also respond to DS. Drought tolerance is a complex trait involving the activation of signaling mechanisms and differentially expressed molecular responses [155].

**Figure 2:**
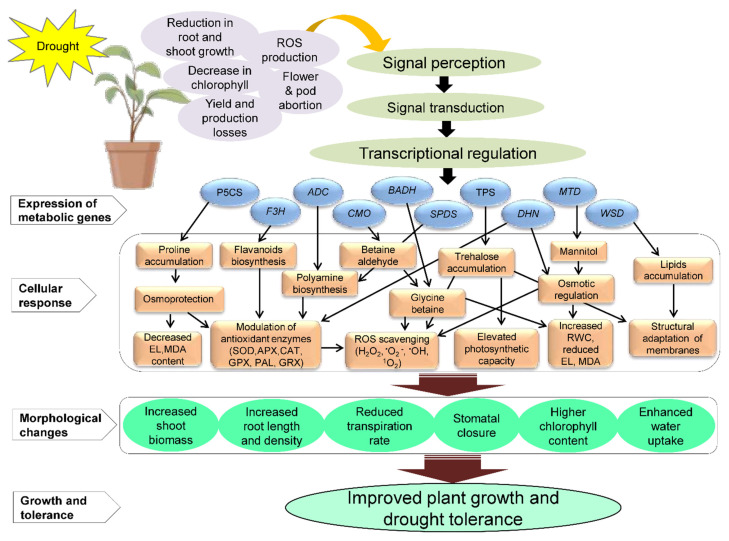
Schematic model displaying drought-induced expression of metabolic genes in transgenic plants. The proposed model depicts drought stress (DS)-mediated (yellow circle) reduction in root and shoot biomass, decrease chlorophyll content, increased reactive oxygen species (ROS) and flower and pod abortion, reducing yield and production (shown in purple oval). Plant DS response and adaptation involve various pathways for signal perception, transduction, transcriptional regulation depicted in olive green ovals, and expression of various metabolic genes shown in aqua color rectangle. Drought-induced expression of metabolic genes, such as *Δ1-pyrroline-5-carboxylate synthetase (P5CS)*, *trehalose-6-phosphate synthase1 (TPS1)*, *dehydrin (DHN), cysteine protease (CP), flavanone 3-hydroxylase (F3H), arginine decarboxylase gene (ADC), choline monooxygenase (CMO), betaine aldehyde dehydrogenase (BADH),* and *spermidine synthase (SPDS*), *mannitol dehydrogenase (MTD)*, *wax synthase/acyl-CoA:diacylglycerol acyltransferase (WSD)* resulted in the accumulation of primary and secondary metabolites. This leads to the accumulation of several osmoprotectants and defensive compounds and ROS detoxification inside cells. Modulation of antioxidants prevents cell damage and maintains homeostasis. SOD, superoxide dismutase; CAT, catalase; APX, ascorbate peroxidase; GPX, guaiacol peroxidase; PAL, phenylalanine ammonia-lyase; GRX, glutaredoxins; MeJA, methyl jasmonate; GB, glycine betain; SA, salicylic acid depicted in orange color rectangles. Morphological changes occurs in plants are shown in light green color rectangles. Plant growth and tolerance are shown in light green color in rectangle. Figure adapted from images created with BioRender.com to draw the proposed model (https://app.biorender.com/biorender-templates (accessed on 18 June 2021)).

Numerous drought-responsive genes have been isolated from various sources, including plants; their characterization for enhancing drought tolerance by developing transgenic plants with increased level of metabolites shown in Table 2. The schematic representation of the proposed model for the application of metabolic genes involved in drought tolerance in plants is shown in Figure 2. It shows the specific responses of metabolic genes under DS in transgenic plants. It also depicts the accumulation of various key metabolites such as glycine betaine, proline, polyamines, trehalose, mannitol, lipids, flavonoids, and other important metabolites in transgenic plants under DS. The accumulation of these metabolites leads to various cellular responses, such as ROS detoxification, modulation of antioxidant activities, increased relative water contents (RWC), decreased electrolytic leakage (EL) and malondialdehyde (MDA) contents, and structural adaptation of membranes, resulting in morphological changes that improve growth and drought tolerance in plants (Figure 2). Targeted metabolites can be enhanced by overexpression of single or multiple genes that produce either direct desired molecule/metabolites or enzymes implicated in the production of the targeted metabolites. The upregulation of these genes resulted in biosynthesis of the metabolite responsible for osmolyte synthesis [156]. The C1A cysteine protease (CysProt) family is one the most abundant proteins, also known as papain-like CysProt). The upregulation of C1A *CysProt* genes is important for protein breakdown during stress responses by reorganizing metabolism, remodeling cell protein compounds, degrading damaged or unnecessary proteins, and remobilizing nutrients [34,157,158]. Gomez-Sanchez et al. [159] reported that drought stimulated the entire C1A CysProt family and upregulates *HvPap-1* and *HvPap*-*19* genes in *H*. *vulgare* leaves. Transgenic *Arabidopsis* overexpressing the *T. aestivum cysteine protease* (*TaCP*) gene showed enhanced drought tolerance and cysteine protease (CP) activity under water-stressed conditions compared to wild-type (WT) plants [160]. 

Overexpression of the *Wax synthase/acyl-CoA:diacylglycerol acyltransferase (WSD1)* gene from Arabidopsis enhanced tolerance to ABA, mannitol, drought and salinity in transgenic *A. thaliana* and *Camelina sativa* plants through increased accumulation of epicuticular wax crystals and higher leaf and stem wax loading [161]. These transgenic plants also had an enhanced recovery rate from drought and salinity stress compared to WT plants. The main symptoms of DS during the vegetative phase include reduced plant height, leaf wilting, and decreased leaf number and area. Reduced plant height under DS is strongly associated with cell enlargement and leaf senescence [162]. Drought stress decreases turgor pressure and photosynthetic rate, decreasing leaf area [163]. Drought stress significantly reduced leaf width and length in *Prunus sargentii* and *Larix kaempferi* [164] and leaf area in *Maclura pomifera* [165], *Triticum aestivum* [166], *Lens culinaris* [167], and *Dracocephalum moldavica* [168]. Plant roots are directly associated with water absorption and play the most significant role in DS [169]. Root system architecture, including root density, root branching, and root hairs, can be affected by DS. For example, maize absorbs more water from dry soil by decreasing lateral root branch density and increasing axial root elongation and rooting depth [170]. 

Dehydrins belong to group II late embryogenesis abundant (LEA) proteins, which are considered stress-responsive proteins involved in forming protective reactions against dehydration in plants [171]. A *dehydrin* gene isolated from *Olea europaea* named *OesDHN* was overexpressed in *Arabidopsis thaliana,* induced by DS, and conferred osmotic stress tolerance [172]. Ectopic expression of *TdDhn-5* gene isolated from *T. durum* showed drought, salt, and osmotic stress tolerance in transgenic *A. thaliana* [173]. Transgenic chickpea (*Cicer arietinum* L.) overexpressing dehydration responsive element binding protein gene from *A. thaliana* (*AtDREB1a*) showed increased tolerance to DS [174]. The leaves of these transgenic lines maintained higher relative water content under soil water deficit which resulted in higher seed yield compared tonon transformed-control. Arginine decarboxylase (ADC) is an essential enzyme responsible for polyamine synthesis under stress conditions. Transgenic *O. sativa* expressing the *ADC* gene from *Datura stramonium* had enhanced drought tolerance, relative to WT plants [175]. The *PtADC* gene from *Poncirus trifoliata* conferred resistance to long-term drought, high osmoticum, dehydration, and cold stress in transgenic *A. thaliana* compared to WT and the mutant, and induced primary root growth [176]. Choline monooxygenase (CMO) catalyzed glycine betaine biosynthesis, a plant osmoprotectant accumulated in response to drought and salinity stress. A plastid-expressed *CMO* gene, namely *BvCMO* isolated from *Beta vulgaris,* showed improved salt and drought tolerance in transgenic *N. tabacum* by accumulating glycine betaine [176]. The *betaine aldehyde dehydrogenase* (*BADH*) gene confers tolerance to abiotic stresses including drought in plants [177]. The *AnBADH* gene from *Ammopiptanthus nanus* conferred drought and high salinity stress tolerance in transgenic *Arabidopsis* [178]. The spermidine synthase (SPDH) enzyme catalyzed spermidine (Spd) synthesis, an important polyamine with low-molecular-weight aliphatic amines that occur ubiquitously in animals, plants, and microorganisms. Overexpression of *Cucurbita ficifolia CfSPDH* gene improved tolerance to drought, chilling, freezing, salinity, hyperosmosis, and paraquat toxicity and modulated expression of various stress-regulated genes in transgenic *A. thaliana* [179]. 

Proline is a compatible osmolyte commonly found in drought-stressed plants. Its accumulation is a typical physiological response to DS in several species [180]. Biosynthesis of proline generally occurs through the glutamate pathway with the help of Δ^1^-pyrroline-5-carboxylate synthase (P5CS) and Δ^1^-pyrroline-5-carboxylate reductase (P5CR) in cytoplasm or chloroplasts [181]. Pyrroline-5-carboxylate (P5C) is an intermediate product of both proline biosynthetic and catabolic processes, synthesized from glutamate by the P5CS enzyme. It is converted to proline by the enzyme P5CR in cytosol and plastids [182]. The *P5CS* gene from various plant sources has been characterized for drought tolerance in different crops, including the *Vigna aconitifolia VaP5CS* gene in transgenic tobacco [183] and *A. thaliana AtP5CS* or *O. sativa OsP5CS* gene in *Petunia hybrida* [184]. Petunia plants transformed with *P5CS* genes, namely *AtP5CS* from *A. thaliana* or *OsP5CS* from *O. sativa*, accumulated proline and exhibited drought tolerance [184]. 

Trehalose is an osmolyte with a vital role in osmotic adjustment [185]. Various genes are involved in the metabolism of trehalose, including the yeast *trehalose-phosphate synthase1* (*TPS1*) gene that has been used to enhance drought tolerance in numerous plant species [186]. The bacterial *trehalose-6-phosphate synthase1* (*ScTPS1*) and *trehalose-6-phosphate synthase2* (*ScTPS2*) genes in *N. tabacum* confer drought tolerance [187] and the *EcTPS* gene in rice enhanced tolerance to drought, salt, and cold stress in transgenic *O. sativa* [188]. Similarly, overexpression of the *O. sativa OsTPS1* gene conferred drought, cold, and salt tolerance in transgenic *O. sativa* [189]. Glucosinolates (GLS) are SMs found in plants of the *Brassicaceae* family, protecting them from herbivory and pathogen attack. The Aux/IAA proteins belong to auxin co-receptors and transcriptional repressors family which have a key role in auxin signaling in plants. The levels of GLS were regulated by the auxin-sensitive Aux/IAA repressors IAA5, IAA6, and IAA19 proteins. These proteins function in a transcriptional cascade that maintains the expression of GLS levels in plants under DS [190]. These Aux/IAA proteins encoded by Aux/IAAgenes IAA5, IAA6, and IAA19 genes which are directly regulated by *DREB2A* and *DREB2B*, transcription factors that are well known for their DS response [191].

**Table 2:** Application of metabolic genes for generating transgenic crops with improved drought tolerance.

Gene	Locus ID	Source	Transgenic Plants	Metabolite Accumulation	Stress Tolerance	References
*Arginine decarboxylase* (*AtADC*)	BT000682	*Arabidopsis thaliana*	*A. thaliana*	Increased putrescine	Drought	[192]
*Arginine decarboxylase* (*DsADC*)	AJ251819	*Datura stramonium*	*Oryza sativa*	Increased putrescine and spermidine	Drought	[193]
*Arginine decarboxylase* (*PtADC)*	HQ008237	*Poncirus trifoliata*	*A. thaliana*	Enhanced putrescine	High osmoticum, dehydration, long-term drought, cold	[175]
*Betaine aldehyde dehydrogenase* (*AnBADH*)	KJ841914	*Ammopiptanthus nanus*	*A. thaliana*	Increased glycine betaine	Drought, salt	[178]
*Chalcone synthase* (*NtCHS*)	LOC107801774	*Nicotiana tabacum*	*N. tabacum*	Increased flavanoids (rutin, quercetin, naringenin)	Drought	[194]
*Choline monooxygenase* (*BvCMO*)	AB221007	*Beta vulgaris*	*N. tabacum*	Increased glycine betaine	Drought, salt	[176]
*Choline oxidase* (*AgcodA*)	AY589052	*Arthrobacter globiformis*	*Solanum tuberosum*	Increased glycine betaine	Water stress	[195]
*Choline oxidase* (*codA*)	AY304485	*A. globiformis*	*S. tuberosum*	Increased glycine betaine	Drought, salt, oxidative	[196]
*Cysteine protease* (*TaCP*)	AY841792	*Triticum aestivum*	*A. thaliana*	Increased *Cysteine protease* activity	Drought	[160]
*Dehydrin* (*OesDHN*)	KR349290	*Olea europaea*	*A. thaliana*	Increased proline	Drought	[172]
*Dehydrin* (*TdDhn*-5)	AY619566	*T. durum*	*A. thaliana*	Increased proline	Drought, salt	[173]
*Dehydrin* (*ShDHN*)	AK319970	*Solanum habrochaites*	*Solanum lycopersicum*	Increased proline	Drought, salt, osmotic stress	[197]
*Dehydrin* (*PmLEAs*)	XM_016796383	*Prunus mume*	*N. tabacum*	Increased proline	Drought, cold	[198]
*Flavanone 3-hydroxylase*(*RsF3H*)	JQ043380	*Reaumuria soongorica*	*R. soongorica*	Increased flavonoids and anthocyanin	Drought, UV-B radiation	[199]
*Mannitol dehydrogenase (CaMTD)*	LOC101510334	*Cicer arietinum*	*C. arietinum*	Increased flavonoids	Drought	[200]
*Mannitol-1-phosphate dehydrogenase* (*EcmtlD*)	EFF7369098	*Escherichia coli*	*T. aestivum*	Increased mannitol	Drought	[201]
*Ornithine* δ-*aminotransferase* (*Atδ*-*OAT*)	NM_123987	*A. thaliana*	*O. sativa*	Increased proline	Drought	[202]
*Ornithine* δ-*aminotransferase* (*OsOAT*)	LOC Os03g44150	*O. sativa*	*O. sativa*	Increased proline	Drought	[203]
*Spermidine synthase* (*CfSPDS*)	BD142348	*Cucurbita ficifolia*	*A. thaliana*	Increased spermidine synthase activity and spermidine content	Drought, chilling, freezing, salinity, hyperosmosis	[179]
*Trehalose-6-phosphate synthase* (*EcTPS*; *otsA*) and *Trehalose-6-phosphate* *phosphatase* (*EcTPP*; *otsB*)	NC_000913	*E. coli*	*O. sativa*	Increased trehalose	Drought, salt, cold	[188]
*Trehalose-6-phosphate synthase1* (*OsTPS1*)	HM050424	*O. sativa*	*O. sativa*	Increased trehalose and proline	Drought, salt, and cold	[189]
*Trehalose-6-phosphatesynthase1* (*ScTPS1*) and *trehalose-6-phosphate synthase2* (*ScTPS2*)	NC_001134	*Saccharomyces cerevisiae*	*N. tabacum*	Enhanced trehalose	Drought	[187]
*Wax synthase/acyl-CoA:diacylglycerol acyltransferase (AtWSD1)*	AT5G37300	*A. thaliana*	*A. thaliana* and *Camelina sativa*	Increased deposition of epicuticular wax crystals and leaf and stem wax loading	Drought	[161]
*WRI4-like gene* (*CeWRI4*)	MW039149	*Cyperus esculentus*	*A. thaliana*	Increased cuticular wax biosynthesis and deposition	Drought	[204]
*Δ1-pyrroline-5-carboxylate synthetase* (*VaP5CS*)	VIRPYRR	*Vigna aconitifolia*	*N. tabacum*	Increased proline	Drought	[183]
*Δ1-pyrroline-5-carboxylate synthetase genes* (*OsP5CS*)	D49714	*O. sativa*	*P. hybrida*	Increased proline	Drought	[184]

Flavonoids are important SMs that play significant roles in maintaining the cellular redox balance of plant cells. Chalcone synthase (CHS) is the key enzyme in the flavonoid biosynthesis pathway and is modulated under DS. Transgenic *N. tabacum* plants overexpressing the *NtCHS* gene showed enhanced drought tolerance and oxidative stress responses under DS, relative to control plants [194]. Transgenic *Brassica napus* overexpressing the BR biosynthesis gene *AtDWARF4* from *Arabidopsis* had improved drought tolerance [205]. Overexpressing *Gossypium hirsutum Gh4CL7* gene enhanced drought stress tolerance in *Arabidopsis* [206]. Drought tolerance is conferred in *N. tabacum* plants through the overexpression of sweet potato *cinnamate 4-hydroxylase* (*IbC4H*), promoting phenolic compound accumulation and increasing the expression of stress-responsive genes [106]. The role of metabolic gene expression for providing drought tolerance in plants is summarized in Table 2. Overall, we conclude that induction of metabolite biosynthesis provides a defensive level of drought protection and enhances growth tolerance in drought-stressed plants.

## 5. Conclusions and Future Research Perspectives

Plants have developed a multifaceted network of defense mechanisms to endure stress environments. Drought is a prevalent unfavorable limiting factor that alters plant growth, development, physiology, metabolism, yield, and production. Plant strategies for drought tolerance include morphological, physiological, and biochemical changes at different phases of plant growth. In the last decade, several metabolic genes have been used to improve drought tolerance in crops using genetic engineering approaches. Metabolic profiling could be useful for characterizing the molecular traits implicated in drought tolerance to provide valuable information for plant breeding programs. Several studies on PMs, SMs, and metabolic genes under DS (e.g., Table 1 and Table 2) revealed valuable information for regulating DS responses. Fulfilling the demand for staple food supply for the increasing human population in coming decades requires awareness of genetically engineered plants and appraisal of their agronomic needs. A possible strategy for enhancing DS tolerance in plants is maintaining the structure and function of cellular components by modulating the expression levels of metabolic genes using genetic engineering. Hence, engineering metabolic genes is one approach for enhancing drought tolerance in crop plants and increasing productivity under DS. Reprogramming PMs or SMs and genetic engineering of metabolic genes will play a key role in plant adaptation and response to DS. Such novel genetic engineering technologies and proposed potential targets of metabolic engineering for drought tolerance will have an enormous impact on sustainable crop yields and productivity to feed the world’s ever-growing population.

## Data Availability

Data presented in this study are available in the article.

## Abbreviations

AAs	Amino acids
DAI	Days after drought imposition
DS	Drought stress
GABA	γ-amino butyric acid
GC-MS	Gas chromatography-mass spectrometry
IAA	Indole-3-acetic acid
NMR	Nuclear magnetic resonance
PMs	Primary metabolites
P5CS	Δ1-pyrroline-5-carboxylate synthase
ROS	Reactive oxygen species
SMs	Secondary metabolites
